# Reconstitution and optimisation of the biosynthesis of bacterial sugar pseudaminic acid (Pse5Ac7Ac) enables preparative enzymatic synthesis of CMP-Pse5Ac7Ac

**DOI:** 10.1038/s41598-021-83707-x

**Published:** 2021-02-26

**Authors:** Harriet S. Chidwick, Emily K. P. Flack, Tessa Keenan, Julia Walton, Gavin H. Thomas, Martin A. Fascione

**Affiliations:** 1 Department of Chemistry, University of York, Heslington Road, York, YO10 5DD UK; 2 Department of Biology, University of York, Heslington Road, York, YO10 5DD UK

**Keywords:** Chemical biology, Carbohydrates, Glycobiology

## Abstract

Pseudaminic acids present on the surface of pathogenic bacteria, including gut pathogens *Campylobacter jejuni* and *Helicobacter pylori*, are postulated to play influential roles in the etiology of associated infectious diseases through modulating flagella assembly and recognition of bacteria by the human immune system. Yet they are underexplored compared to other areas of glycoscience, in particular enzymes responsible for the glycosyltransfer of these sugars in bacteria are still to be unambiguously characterised. This can be largely attributed to a lack of access to nucleotide-activated pseudaminic acid glycosyl donors, such as CMP-Pse5Ac7Ac. Herein we reconstitute the biosynthesis of Pse5Ac7Ac in vitro using enzymes from *C. jejuni* (PseBCHGI) in the process optimising coupled turnover with PseBC using deuterium wash in experiments, and establishing a method for co-factor regeneration in PseH tunover. Furthermore we establish conditions for purification of a soluble CMP-Pse5Ac7Ac synthetase enzyme PseF from *Aeromonas caviae* and utilise it in combination with the *C. jejuni* enzymes to achieve practical preparative synthesis of CMP-Pse5Ac7Ac in vitro, facilitating future biological studies.

## Introduction

**Figure 1:**
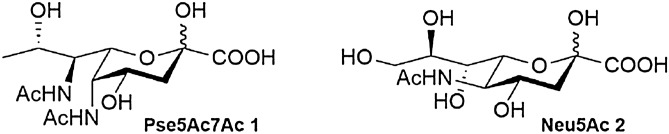
The nonulosonic acids; Pse5Ac7Ac **1** and Neu5Ac **2**.

Pseudaminic acids are non-mammalian nonulosonic acid sugars present in glycoconjugates on the surface of a number of pathogenic bacteria^1,2^, including multidrug resistant *Pseudomonas aeruginosa*^3–6^ and *Acinetobacter baumannii*^7–9^, and gut pathogens *Campylobacter jejuni*^10–12^ and *Helicobacter pylori*^13^. The parent sugar α-5,7-diacetamido-3,5,7,9-tetradeoxy-l-glycero-l-manno-non-2-ulosonic acid, or Pse5Ac7Ac **1** (often referred to as simply pseudaminic acid, or 5,7-diacetyl pseudaminic acid)^1,2^, shares structural similarities with the widely known sialic acid Neu5Ac **2**^14,15^, but is relatively understudied in comparison (Fig. 1). For example, glycosyltransferase enzymes that catalyse the attachment of pseudaminic acid sugars to proteins or glycans are yet to be unequivocally characterised despite mounting evidence that pseudaminic acid glycosylation of flagella is important for autoagglutination and bacterial mobility in both *C. jejuni*^16^ and *H. pylori*^13^ and may also modulate the host’s immune response to bacterial infection^17^. A major reason for this relative lack of progress is that chemical access to pseudaminic acid derivatives such as nucleotide-activated cytidine monophosphate (CMP)-Pse5Ac7Ac **3**, required for the study of Leloir glycosyltransferases, is challenging due to their complex structural architecture^18^.

**Scheme 1:**
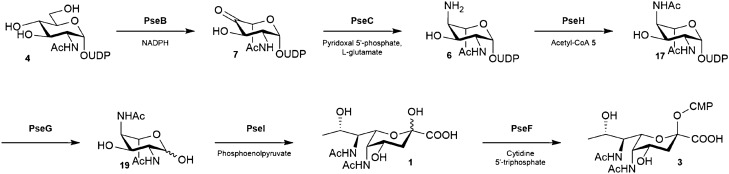
*H. pylori*/*C. jejuni* biosynthetic pathway from UDP-GlcNAc **4** to CMP-Pse5Ac7Ac **3**^19^.

Exploiting Pse5Ac7Ac biosynthetic pathways from bacteria offers an enticing alternative to overcome this challenge, particularly as the biosynthesis of CMP-Pse5Ac7Ac **3** is well established in *C. jejuni* and *H. pylori*. Indeed the *H. pylori* biosynthetic enzymes PseB (a dehydratase/epimerase), PseC (an aminotransferase), PseH (an acetyl transferase), PseG (a nucleotidase), PseI (a Pse5Ac7Ac synthase) and PseF (a CMP-Pse5Ac7Ac synthetase) have previously been coopted for the in vitro synthesis of the glycosyl donor **3** from UDP-GlcNAc **4** starting material (Scheme 1)^19^. However this purely enzymatic workflow has yet to be adopted by the glycoscience community at large as a practical route to CMP-Pse5Ac7Ac **3**. Herein we examine the in vitro reconstitution of the *C. jejuni* Pse5Ac7Ac biosynthetic pathway, and use negative ion LCMS analysis to optimise the workflow. In the process we minimise byproduct formation in the coupled transformation catalysed by PseB/C^20^, and establish a method for regeneration of the expensive co-factor acetyl-coenzyme A **5** (Ac-CoA) in the PseH catalysed step, which increases the practical and economic viability of the enzymatic process. In addition we purify a soluble active PseF enzyme from *Aeromonas caviae* enabling the preparative synthesis of CMP-Pse5Ac7Ac **3** on a multi-milligram scale.

## Results

### Pseudaminic acid biosynthetic enzyme production

**Figure 2:**
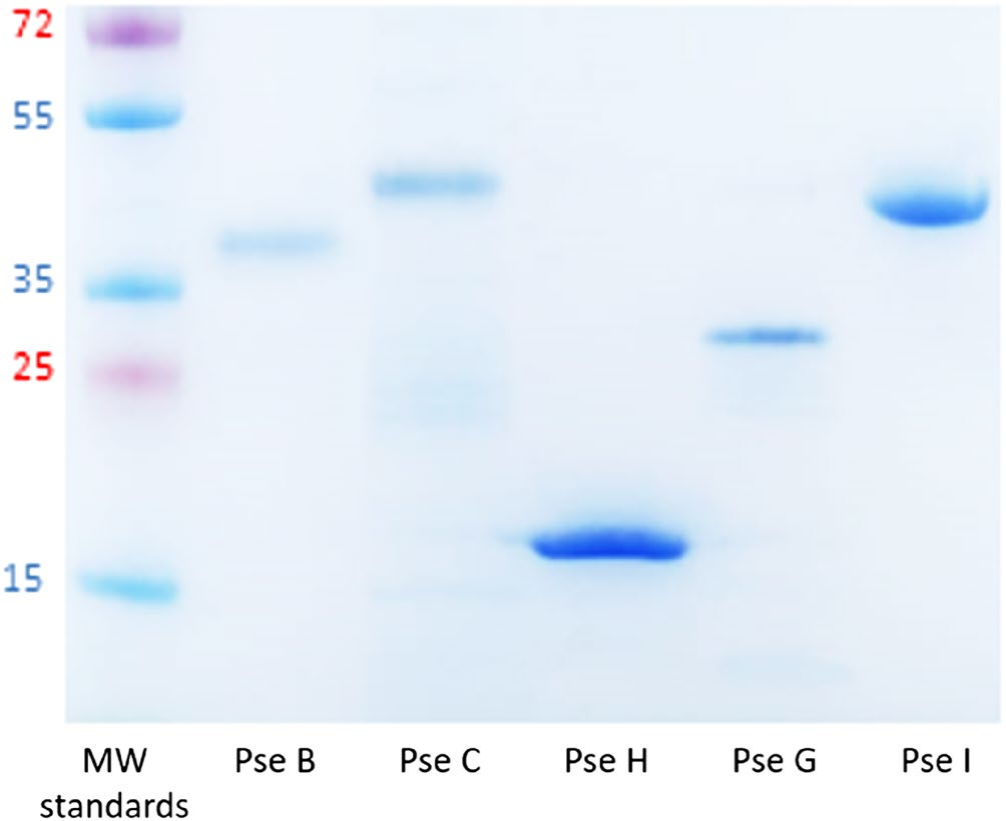
4–20% SDS-PAGE of Ni^2+^-His_6_ purified *C. jejuni* Pse5Ac7Ac biosynthetic enzymes PseBCHGI.

Cognisant that scaleable in vitro enzymatic synthesis of CMP-Pse5Ac7Ac **3** would be dependant on ready access to the six biosynthetic enzymes, PseBCHGIF (Scheme 1)^20^, we initially set out to attain the large scale production of the recombinant enzymes. Expression trials in *E. coli* BL21 (DE3) cells, using plasmids encoding N-terminal His-tagged *PseC*, *PseH*, *PseG*, *PseI*, and C-terminal His-tagged *PseB* genes from *C. jejuni*, imaged on SDS-PAGE displayed overexpressed enzymes at the predicted molecular weight for the desired enzyme and allowed for identification of induction conditions (Table 1, Supplementary Fig. SI.1). The production of the PseB, PseH, PseG and PseI enzymes routinely afforded mg/L yields of protein post-purification, greater or equal to those previously reported (Table 1, Fig. 2)^20^. However, the PseC enzyme displayed a propensity to precipitate during purification when expressed at an induction temperature of 37 °C over 4 h. We therefore explored reducing the temperature to 16 °C, which reduced the concentration of protein produced but also precipitation post purification. These conditions were therefore used in future large scale protein preparations. Unfortunately the N-terminal His-tagged *H. pylori* PseF enzyme was largely insoluble in all expression conditions trialled in our hands (Supplementary Fig. SI.2), we therefore turned our attention to the PseF homologue from *A. caviae*, a gram negative bacterium which presents Pse5Ac7Ac **1** on its flagella^21^. Expression of *A. caviae* PseF was investigated in *E. coli* BL21 (DE3) cells with induction conditions of 0.1 mM IPTG followed by three hours incubation at 30 °C found to yield soluble protein at ~13 mg L^−1^ post purification. The enzyme was characterised by mass spectrometry, circular dichroism and size exclusion chromatography-multi-angle laser light scattering (SEC-MALS) (Supplementary Fig. SI.4–6), which confirmed it existed as a homodimer, consistent with homologous CMP-Neu5Ac synthetase^22^ and CMP-Kdo synthetase^23^. Furthermore the activity of the enzyme as a CMP-Pse5Ac7Ac synthetase was confirmed in small scale negative ion ESI-LCMS assays with Pse5Ac7Ac **1** and CTP (Supplementary Fig. SI.7). Notably all enzymes could be stored in the freezer without cryoprotectant prior to use.

**Table 1:** Conditions used in the induction of Pse5Ac7Ac biosynthetic enzymes and the resulting quantity of enzyme.

	Molecular weight/kDa	IPTG conc^n^/mM	Induction temp/°C	Induction time/h	Enzyme quantity/mg L^−1^
PseB	37.4	0.1	37	4	14
PseC	42.3	0.1	16	4	9
PseH	18.7	0.1	16	20	17
PseG	31.3	0.5	37	4	11
PseI	38.6	0.5	37	20	18

### Optimising the PseB/PseC coupled transformation of UDP-GlcNAc

**Figure 3:**
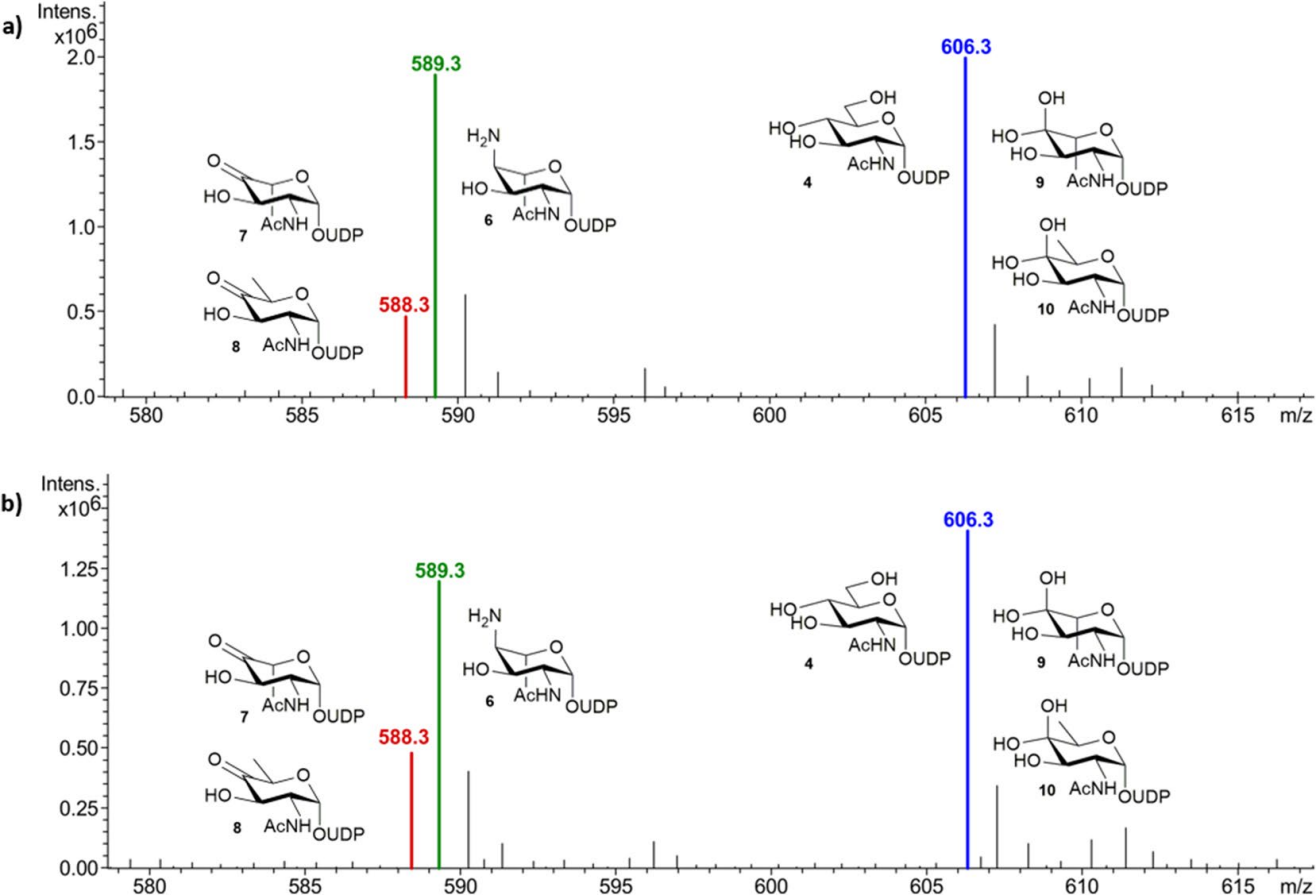
*C. jejuni* PseB and PseC reaction progression (**a**) after 45 min of incubation with UDP-GlcNAc **4**, and (**b**) after 6 h of incubation with UDP-GlcNAc **4** showing no significant further reaction progress to the PseC product **6**.

**Scheme 2:**
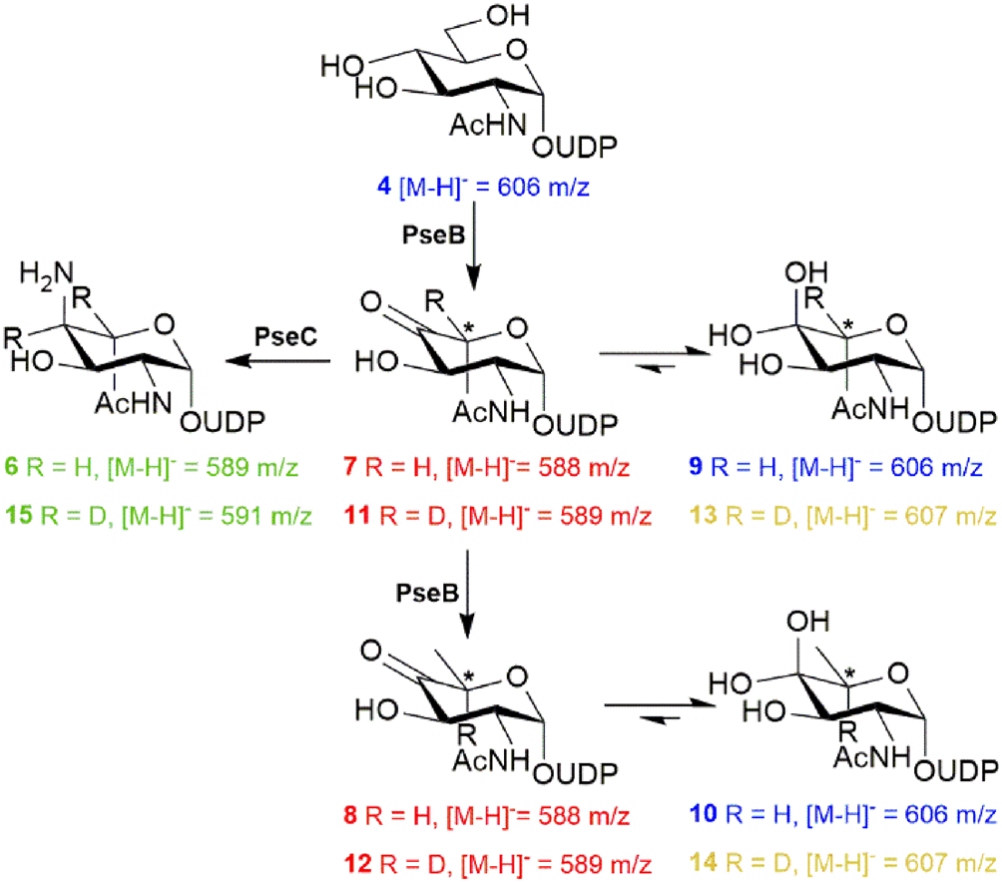
Full *C. jejuni* PseB and PseC catalysed reactions in water or deuterated buffer.

**Figure 4:**
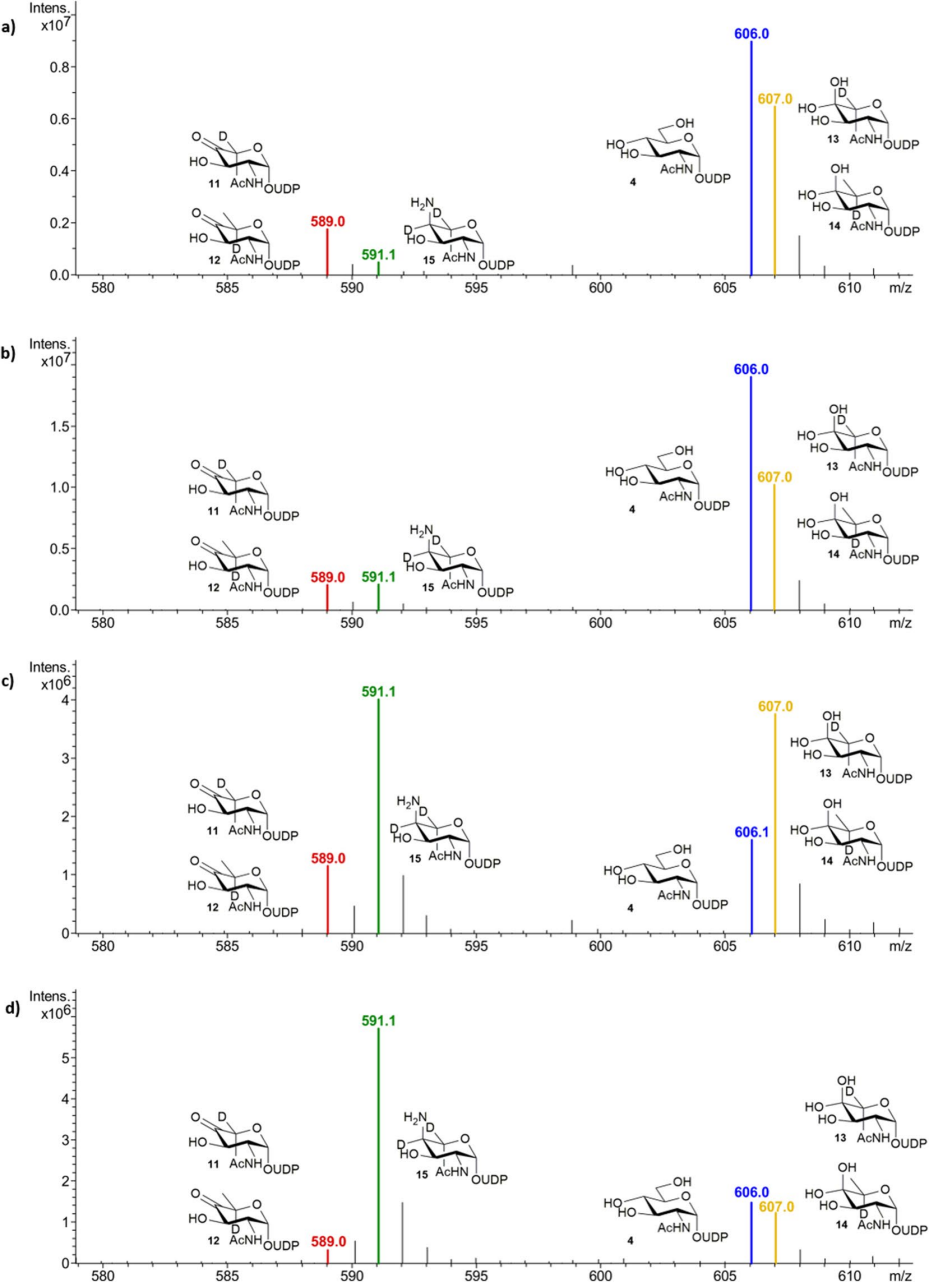
Negative ESI LC–MS monitoring relative conversion to the PseB and PseC products in deuterated buffer with UDP-GlcNAc **4** (**a**) after 10 min with equal concentrations of PseB and PseC, (**b**) after 10 min with a PseB:PseC concentration of 1:5, (**c**) after 2 h with equal concentrations of PseB and PseC or (**d**) after 2 h with a PseB:PseC concentration of 1:5.

With the Pse5Ac7Ac biosynthetic enzymes in hand, we initially set out to explore the use of PseB and PseC in the first coupled transformation of the UDP-GlcNAc **4** starting material to afford PseC product UDP-4-amino-4,6-dideoxy-β-l-AltNAc **6** (Scheme 1). This intermediate had previously proved valuable as an access point to obtaining unnaturally acylated Pse5Ac7Ac precursors by chemical methods^24^, and also enables characterisation of the third enzyme in the pathway PseH^25^, a potential target for inhibitor studies. However, a reaction using the *C. jejuni* enzymes and respective co-factors with 50 mg UDP-GlcNAc **4** (in negative ion ESI-LCMS analysis [M−H]^−^ = 606.3 *m/z*, blue, consistent with the mass of UDP-GlcNAc, 607.35), only yielded a 42% conversion to the desired UDP-4-amino-4,6-dideoxy-β-l-AltNAc **6** ([M−H]^−^ = 589 *m/z*, green) by negative ion ESI-LCMS analysis (Fig. 3). Significant apparent starting material peak at 606 *m/z* also remained after 45 min, in addition to some seemingly unreacted PseB product UDP-4-keto-6-deoxy-β-l-IdoNAc **7** ([M−H]^−^ = 588 *m/z*, red), with no increase in conversion noted over 6 h. This result therefore implied turnover by the PseB enzyme may be the limiting step in the coupled transformation, however we found increasing the PseB enzyme concentration had no effect on conversion. Consideration of the PseB mechanism in more detail, and noting previous biochemical characterisation, revealed that in addition to acting as a 5-inverting 4,6-dehydratase, PseB can also catalyse a further C5 epimerisation (highlighted with an asterisk in Scheme 2) of the initial product **7** to afford UDP-4-keto-6-deoxy GlcNAc **8**^20,26^, albeit at a lower rate. The GlcNAc configured **8** is the first intermediate en route to the biosynthesis of the bacterial sugar UDP-diNAcBac, integral to N-linked protein glycosylation in *C. jejuni* and no longer a substrate for PseC in the Pse5Ac7Ac pathway^25,27^. We hypothesised that our transformation might have stalled due to C5-epimerisation, and that the epimeric ketone products of PseB **7** and **8** (with identical ESI-LCMS peaks at [M−H]^−^ = 588 *m/z*, red) would exist in equilibrium with their hydrated counterparts **9** and **10**^26^. This would complicate ESI analysis as the hydrates would have the same [M−H]^−^ peak (606 *m/z*) as the UDP-GlcNAc **4** starting material, therefore the apparent remaining starting material in the PseB/C coupled reaction could instead represent epimeric hydrated PseB products **9** and **10**. This suggested an excess of PseC enzyme rather than PseB may be required to drive the reaction forward prior to epimerisation occurring. To explore this hypothesis however it was necessary to clarify the ESI-LCMS analysis of the reaction and distinguish between the intermediates in the reaction, particularly hydrates **9** and **10** and the UDP-GlcNAc starting material **4**. We therefore opted to perform the reaction in deuterated buffer, as during the PseB catalysed transformation incorporation of a non-exchangeable C5-proton from bulk solvent occurs^26^, as well as incorporation of a further non-exchangeable C4-proton from bulk solvent in the PseC catalysed transformation (Supplementary Scheme SI.1 and SI.2)^28^. Thus in deuterated buffer we expected the [M−H]^−^ peak in ESI-LCMS spectra (Fig. 4) of the UDP-GlcNAc starting material **4** to be unaffected ([M−H]^−^ = 606 *m/z*, blue), but the peaks to increase by 1 *m/z* for the PseB ketone products **11** and **12** ([M−H]^−^ = 589 *m/z*, red) and their respective hydrates **13** and **14** ([M−H]^−^ = 607 *m/z*, yellow), and the peak to increase by 2 *m/z* for the PseC product **15** ([M−H]^−^ = 591 *m/z*, green). Although all other exchangeable protons of the hydroxyl groups would also become deuterated following incubation in D_2_O potentially complicating analysis we anticipated that an excess of H_2_O in the mobile phase during ESI-LCMS would result in re-exchange of these hydroxyl groups, thus enabling LCMS to be used to compare the relative conversions to the PseC product at different time points and ratios of PseB and PseC. Indeed when UDP-GlcNAc **4** was incubated with either a 1:1 or 1:5 ratio of PseB and PseC in deuterated sodium phosphate buffer for 10 min (Fig. 4a,b), the presence of both UDP-GlcNAc starting material **4** ([M−H]^−^ = 606 *m/z*, blue), and C5-deuterated PseB hydrated product **13** and **14** ([M−H]^−^ = 607 *m/z*, yellow) was apparent, with an increase in the relative PseC concentration seemingly having little affect on the progress of the PseB catalysed reaction. However after 2 h using a 1:1 ratio of PseB:PseC (Fig. 4c) significant C5-deuterated PseB products remained (48% of the total biosynthetic intermediates observed in ESI-LCMS) with 38% conversion to C5,C4-dideuterated PseC product **15** ([M−H]^−^ = 591 *m/z*, green) observed, whilst increasing the relative concentration of PseB:PseC to 1:5 over 2 h (Fig. 4d) resulted in an increased 66% conversion to C5,C4-dideuterated PseC product, with only 20% C5-deuterated PseB products remaining, and no change after 24 h. Thus implying when using equimolar concentrations of PseB and PseC enzymes in vitro, non-productive PseB catalysed C5 epimerisation can compete with productive PseC turnover reducing overall enzymatic conversion. However, increasing the concentration of PseC with respect to PseB drives the coupled transformation forward, minimising the formation of C5 epimeric byproduct. Notably in a one-pot multienzyme synthesis of Pse5Ac7Ac **1**, subsequent turnover of the PseC product by the next enzyme in the biosynthetic pathway (PseH) would likely further increase flux through the productive PseC pathway^19^, however the optimised ratio of PseB: PseC determined here would be particularly useful in the absence of PseH i.e. when isolated PseC product UDP-4-amino-4,6-dideoxy-β-l-AltNAc **6** is the desired target, and or when a non-enzymatic acylation procedure is utilised to install natural or unnatural N-acyl groups^24^.

### Optimising PseH turnover in vitro through co-factor regeneration

The third step in the Pse5Ac7Ac biosynthesis is the transfer of an acetyl group to *N*4 of the PseC product **6** affording UDP-4-acetamido-4,6-dideoxy-β-l-AltNAc **17** (Scheme 1). Enzymatically this transformation is catalysed by PseH^29,30^, an aminoglycoside *N*-acetyl transferase from the GNAT superfamily, and as such utilises Ac-CoA **5** as a co-factor^31^. Despite being used ubiquitously as an acetyl transfer group in vivo, the complex structure of Ac-CoA **5** makes it a high cost reagent, limiting its use for in vitro preparative enzymatic synthesis of Pse5Ac7Ac **1**. Therefore to make the synthesis more economically viable, we considered strategies to reduce the amount of Ac-CoA **5** required in the acylation of PseC product **6**. Notably chemical acetylation has been used previously to complete this transformation, and offers an enticing opportunity to install unnatural functionality into the Pse5Ac7Ac **1** backbone^18,24,25^. However the components of such chemical acetylation reactions are invariably incompatible with subsequent enzymatic transformations, necessitating extra purifications of biosynthetic intermediates. Therefore, instead we sought to explore a method for recycling Ac-CoA **5** which would be compatible with a one-pot multienzymatic synthesis of Pse5Ac7Ac **1** and its derivatives.

**Scheme 3:**
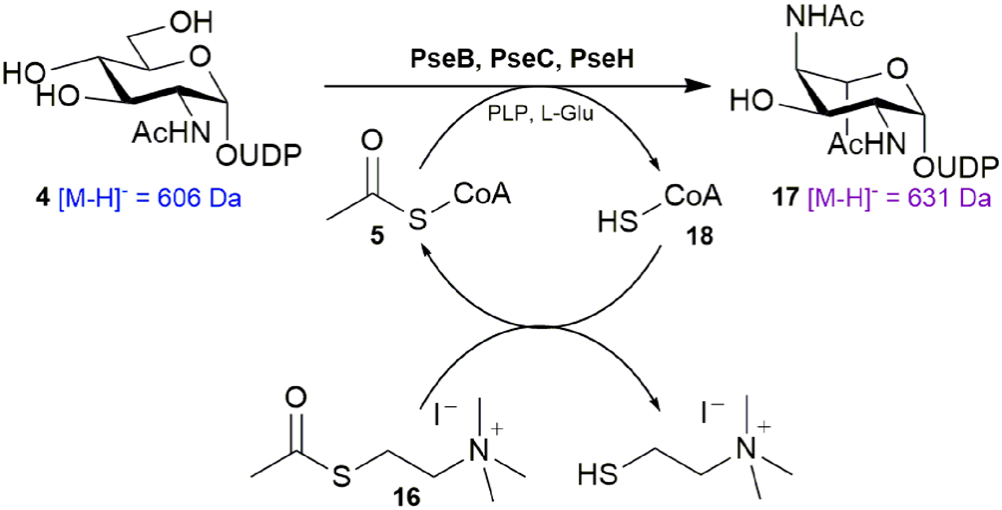
In situ regeneration of the acetyltransfer co-factor **5**, with acetylthiocholine iodide **16**, during the one-pot three enzyme synthesis of the Pse5Ac7Ac biosynthetic intermediate **17**.

**Figure 5:**
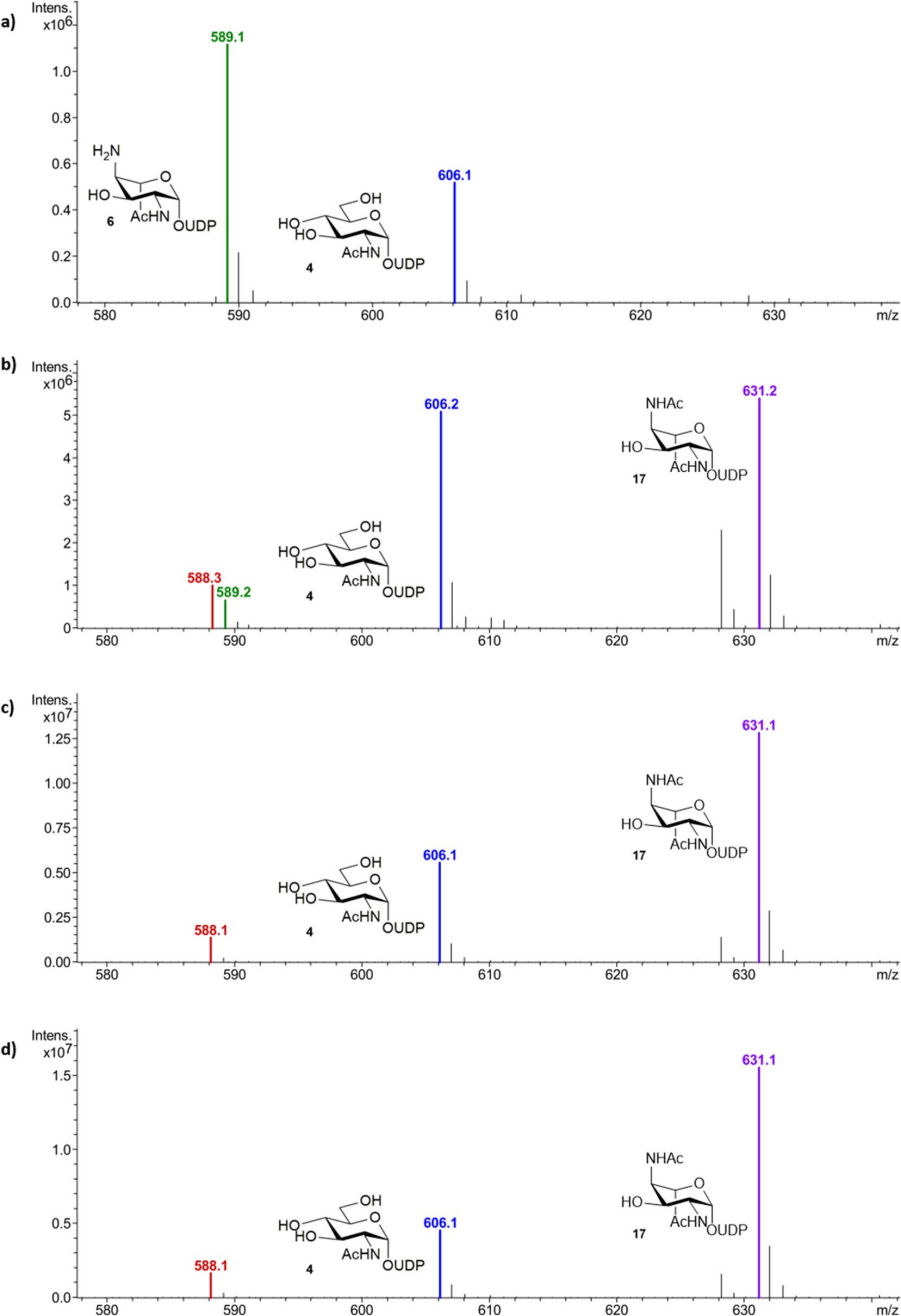
Negative ESI LC–MS analysis of relative conversion to the PseH product **17** from UDP-GlcNAc **4**, investigating the use of acetylthiocholine iodide **16** as a regeneration factor with sub-stoichiometric amounts of Ac-CoA **5** (**a**) 0 mM Ac-CoA **5** and 20 mM acetylthiocholine iodide **16**, (**b**) 0.15 mM Ac-CoA **5** and 0 mM acetylthiocholine iodide **16**, (**c**) 0.15 mM Ac-CoA **5** and 2 mM acetylthiocholine iodide **16**, and (**d**) 0.15 mM Ac-CoA **5** and 20 mM acetylthiocholine iodide **16**.

Previously the thioester acetyl thiocholine iodide **16** had been reported as a low cost acetyl transfer agent in the regeneration of sub-stoichiometric amounts of Ac-CoA **5** for the synthesis of citric acid^32^. We therefore opted to apply this system for recycling Ac-CoA **5** in the PseH catalysed acetylation of **6**, wherein any catalytic CoA thiol **18** liberated after acetylation would undergo in situ thioester exchange with the water soluble acetyl thioester **16**, regenerating the co-factor **5** (Scheme 3). In order to ascertain the efficiency of this method a number of small scale three-enzyme one-pot reactions were set up with UDP-GlcNAc **4** (1 mM), PseB (25 μM), PseC (125 μM), PseH (50 μM), and either sub-stoichiometric Ac-CoA **5** (0.15 mM) and increasing concentrations of acetyl thiocholine iodide **16** (0–20 mM) or Ac-CoA **5** (0 mM) and acetyl thiocholine iodide **16** (20 mM) as a control. The reactions were once more monitored by negative ESI-LCMS and the relative conversion to acetylated PseH product **17** ([M−H]^−^ = 631 *m/z*, purple) calculated as a percentage of the total biosynthetic intermediates remaining. In the absence of any Ac-CoA **5** and 20 mM acetyl thiocholine iodide **16** (Fig. 5a), no PseH product **17** is observed, indicating that **16** cannot itself act as a co-factor for the reaction. Addition of sub stoichiometric Ac-CoA **5** (0.15 mM) alone does yield PseH product **17** (Fig. 5b) but as expected at a lower overall conversion. However this conversion could be increased to 66% upon addition of 2 mM acetyl thiocholine iodide **16** (Fig. 5c), and 72% when **16** was included at 20 mM (Fig. 5d), indicating regeneration of the catalytic Ac-CoA **5** does occur through in situ thioester exchange. Indeed even using lower cost sub-stoichiometric CoA thiol **18** at 0.15 mM, as opposed to Ac-CoA **5** could also yield 65% conversion to the PseH product **17** in the presence of 20 mM **16**, and a conversion of 44% could still be achieved at 0.0015 mM CoA thiol **18** (Supplementary Fig. SI.8a). Similarly increasing the concentration of acetyl thiocholine iodide **16** from 20 to 100 mM, in the presence of 0.0015 mM CoA thiol **18** resulted in an increased 61% conversion to the PseH product **17** (Supplementary Fig. SI.8b). These conditions represent a 1000 fold decrease in the level of Ac-CoA **5**/CoA **18** previously required for PseH turnover^19^.

### Optimised ‘one-pot’ multienzyme preparative synthesis of CMP-Pse5Ac7Ac 3

**Scheme 4:**
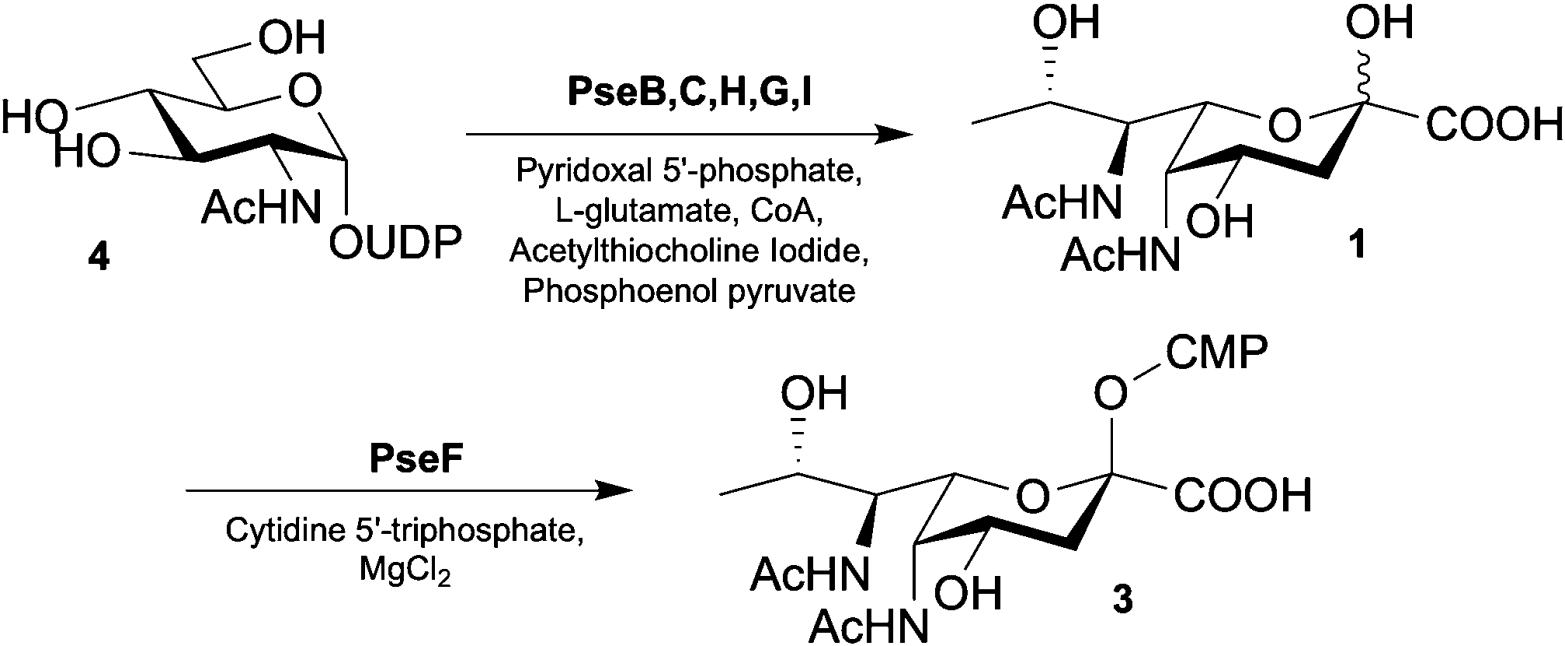
“One-pot” chemoenzymatic synthesis of CMP-Pse5Ac7Ac **3** from UDP-GlcNAc **4** using the biosynthetic enzymes under optimised in vitro conditions.

The vast reduction in co-factor requirement and cost for PseH turnover, allied to the optimisation of the PseB/C coupled transformation now made a ‘one-pot’ two-step multienzyme synthesis more economically viable and practical for production of activated CMP-Pse5Ac7Ac **3**. To demonstrate, we completed the preparative scale synthesis and purification of CMP-Pse5Ac7Ac **3** using the optimised conditions starting from 90 mg UDP-GlcNAc **4** (Scheme 4). We utilised the purified *C. jejuni* enzymes PseB, PseC (in excess), PseH (using Ac-CoA **5** regeneration), PseG which hydrolyses the UDP group, and the Pse5Ac7Ac synthase PseI which condenses phosphoenolpyruvate (PEP) with the newly formed reducing terminus in the PseG product **19**, to afford Pse5Ac7Ac **1** in one-pot over 12 h. Subsequently, the newly characterised soluble CMP-Pse5Ac7Ac synthetase PseF from *A. caviae*, was added to the mixture catalysing conversion to the activated Leloir glycosyl donor CMP-Pse5Ac7Ac **3**.

## Discussion

In order to optimise the in vitro reconstitution of the biosynthesis of the bacterial nonulosonic acid sugar Pse5Ac7Ac **1** we have explored the relationship between the transformations catalysed by the first two enzymes in the biosynthetic pathway from *C. jejuni*, PseB and PseC. Notably PseB catalyses an undesired secondary epimerisation, which poses a challenge for in vitro enzymatic synthesis of Pse5Ac7Ac **1** as the resulting epimeric product is no longer a substrate for PseC^20,26^, but rather the PglE enzyme in UDP-diNAcBac biosynthesis, a precursor to *N*-linked glycoproteins in *C. jejuni*^25,27^. Although the enzymatic epimerisation reaction has been previously disclosed, the optimisation of the coupled PseB/C reaction to maximise flux through the Pse5Ac7Ac pathway was unexplored. We have unequivocally demonstrated that deuterium wash-in experiments enable optimisation of comparative PseB and PseC enzyme concentrations for this transformation and thus maximise desired conversion in ESI-LCMS experiments. It would be beneficial in further investigations to focus on determining the residues involved in the PseB catalysed epimerisation to reduce the need for excess PseC enzyme. Indeed previous mutagenesis studies have highlighted PseB active site residues which are essential for the initial inversion and dehydration but seemingly play no role in the secondary epimerisation^26^, thus implying that rational mutagenesis studies may be used to eliminate undesired byproduct formation with miminal effect on the rate of the desired transformation.

Furthermore in the third step in Pse5Ac7Ac biosynthesis, PseH catalysed acetylation of the 4-amino group, we established a method for in situ regeneration of the expensive co-factor Ac-CoA **5** using acetyl thiocholine iodide **16** as an acetyl transfer reagent. This advance significantly increases the economic viability of in vitro enzymatic synthesis of Pse5Ac7Ac derivatives, and eliminates the need for multiple purification steps as is required when chemical acetylation is employed.

We showcased the benefits of these optimisation studies by combining PseB, PseC and PseH with the final two steps in the Pse5Ac7Ac pathway PseG and PseI, in the process establishing standard conditions for large scale production and storage of the enzymes. The CMP-Pse5Ac7Ac synthetase PseF from *H. pylori 26695* has previously^19^ been purified so we were curious as to why the majority of the expressed protein remained insoluble in our hands. Consideration of the constructs physiochemical parameters identified that, although not classified as hydrophobic (GRAVY score = − 0.34)^33^, the resulting protein sequence was classified as unstable in vitro with an instability index of 45.3. Therefore our attention turned to other CMP-Pse5Ac7Ac synthetases such as the enzyme encoded by *Cj1311* from *C. jejuni* 81-176^34^ and a putative enzyme in *A. caviae* which both share similar sequence identity with *Hp*PseF, 37.2% and 35.9% respectively (aligned in Clustal Omega). All three proteins lack transmembrane regions^35^ and are predicted to be cytoplasmic^36^, which is concordant with their negative GRAVY scores (*Cj*PseF − 0.32 and *Ac*PseF − 0.22). However considering their function as carbohydrate-active enzymes they may be membrane associated with Pse5Ac7Ac **1** activation occurring in the cytoplasm near the inner membrane prior to utilisation by Pse5Ac7Ac glycosyltransferases. The instability index of *Cj*PseF was calculated as 53.6 and hence predicted to be even less stable than the *H. pylori* counterpart. Therefore we focussed on *Ac*PseF^21^ as it has the lower instability index (40.4) and successfully purified soluble protein obtaining a yield of 13 mg L^−1^. Preliminary characterisation of *Ac*PseF using SEC-MALS confirmed it existed predominantly (> 99%) as a homodimer in solution, and ESI-LCMS studies confirmed its activity as a bona fide CMP-Pse5Ac7Ac synthetase, with further biochemical characterisation a subject of future work. Additionally CD studies of the protein indicated it may also be amenable to crystallisation with over 86% secondary structure, consistent with the computational data obtained for homologous *Hp*PseF^37^. *Ac*PseF also shows 27% sequence identity to the CMP-Neu5Ac synthetase from *Neisseria meningitidus* (*Nm*CNS), for which a 2 Å X-ray crystal structure has been solved with the substrate analogue CDP present in the active site and Neu5Ac **2** docked^36^. Unsurprisingly alignment of these sequences alongside *Hp*PseF and *Cj*PseF, in addition to CMP-Kdo synthetase homologues^22,23,38,39^ revealed conservation of several key residues, such as those involved in binding to the cytosine moiety. However differences in the sequence between *Ac*PseF and *Nm*CNS at residues predicted to bind the NHAc substituent at C5, which is equatorial in Neu5Ac **2**, as opposed to axial in Pse5Ac7Ac **1**, and the residues which are proposed to bind the C6 propyl chain in Neu5Ac **2** are also apparent. These differences in sequence may in-part account for the altered specificity for carbohydrate substrates between these enzymes. Importantly, we further demonstrated that the soluble *Ac*PseF enzyme was suitable for a “one-pot” multienzymatic synthesis with the biosynthetic enzymes from *C. jejuni*, which enabled the preparative synthesis of purified CMP-Pse5Ac7Ac **3** from UDP-GlcNAc **4**. With multimilligram quantities of the activated Leloir glycosyl donor now in hand and practically accessible, future structural studies of PseF enzymes and perhaps more crucially biochemical characterisation of elusive Pse5Ac7Ac glycosyltransferases are eminently more feasible.

## Methods

### General methods

Negative ESI LC–MS was carried out on a high performance Dionex UltiMate 3000 LC system (Thermo Scientific) fitted with a Waters CORTECS T3 column (2.7 μm, 150 × 2.1 mm) and linked to a Bruker HCTultra ETD II system (Bruker Daltonics) MS, using a 30–70% gradient of MeCN (0.1% formic acid) in H_2_O (0.1% formic acid) over 12 min.

1D and 2D NMR spectra were recorded on a Bruker Avance Neo 700 MHz spectrometer.

### *C. jejuni* PseB, C, H, G, I

PseB (WP_002869093.1) pET-30a and PseC (WP_002856503.1) pFO4 recombinant plasmids^1^ were electroporated into *E. coli* BL21(DE3) cells and pET-15b vectors containing PseH (WP_002781802.1), PseG (WP_002830499.1) or PseI (WP_002870258.1) (purchased from GenScript, restriction enzymes NdeI and BamHI) chemically transformed into *E. coli* BL21(DE3). For expression trials, cells were streaked onto LB agar containing appropriate antibiotics and incubated (37 °C, overnight) before inoculation of LB with a single colony and incubation (180 rpm, 37 °C, overnight). 2 mL of culture was added per litre of media and incubated (180 rpm, 37 °C) until an OD_600_ of 0.6 was reached whereby aliquots of culture were subjected to different induction conditions. Cell pellets were collected via centrifugation (10,000×*g*, 10 min, 6 °C) and resuspended in BugBuster containing protease inhibitor tablets and treated as per the manufacturer’s instructions. Insoluble material was removed following centrifugation (10,000×*g*, 10 min, 6 °C) and the supernatant analysed via SDS PAGE (Supplementary Fig. SI.1). Large scale protein expression was carried out as above using the optimised inductions conditions as discussed in the main text and cell pellets were collected via centrifugation (6000×*g*, 40 min, 6 °C) and stored at − 80 °C until required. Cell pellets were resuspended in cold lysis buffer (50 mM sodium phosphate buffer, pH 7.4, 400 mM NaCl, 10 mM imidazole, Benzonase (25 U/L media), protease inhibitor tablet) and sonicated. The supernatant following centrifugation (20, 000×*g*, 20 min, 6 °C) was loaded onto a HisTrap HP Ni^2+^ affinity column pre-equilibrated with 50 mM sodium phosphate buffer, pH 7.4, 400 mM NaCl, 10 mM imidazole. After washing (7 C.V) with the same buffer, a linear gradient of 10 mM to 300 mM imidazole was applied (15 C.V) and fractions containing desired protein were desalted into 25 mM sodium phosphate buffer, pH 7.4, 50 mM NaCl.

### *H. pylori* PseF

*H. pylori* PseF (WP_001201444.1) pET-15b recombinant plasmid was electroporated into *E. coli* BL21(DE3) and *E. coli* Turner (DE3) cells, streaked onto LB_Amp_ agar and incubated (37 °C, overnight). A single colony of *E. coli* BL21(DE3) cells was used to inoculate 2xYT_Amp_ (60 mL) and incubated (37 °C, 180 rpm, overnight). The culture was further diluted with 2xYT_Amp_ (4 L) and incubated (30 °C, 180 rpm) until an OD_600_ of 0.6 was reached whereby Isopropyl-β-d 1-thiogalactopyranoside (IPTG) was added to a final concentration of 0.1 mM and further incubated (37 °C, 180 rpm, 2.75 h). Cell pellets were collected via centrifugation (6000×*g*, 30 min, 4 °C), resuspended in cold lysis buffer supplemented with 1 mM MgCl_2_ and 10 mM β-mercaptoethanol and sonicated. The supernatant following centrifugation (17,700×*g*, 35 min, 4 °C) was loaded onto a HisTrap HP Ni^2+^ affinity column pre-equilibrated with 50 mM sodium phosphate buffer, pH 7.3, 400 mM NaCl, 10 mM β-mercaptoethanol, and 10 mM imidazole. After washing (10 C.V) with the same buffer, a linear gradient of 10–250 mM imidazole was applied (30 C.V) followed by 500 mM imidazole (10 C.V’s) and fractions were analysed via SDS PAGE (Supplementary Fig. SI.2). For expression trials, a single colony from *E. coli* BL21(DE3) and *E. coli* Turner (DE3) cells transformed with the PseF recombinant plasmid were used to inoculate 2xYT_Amp_ and incubated (37 °C, 180 rpm, overnight), before being diluted in 2xYT_Amp_ to an OD_600_ of 0.02 and incubated (180 rpm, 37 °C). Aliquots of culture were subjected to different induction conditions and cell pellets collected via centrifugation (10,000×g, 10 min, 6 °C). Cell pellets were resuspended in 50 μL lysis buffer (as above) supplemented with 1 mg mL^−1^ lysozyme and incubated (37 °C, 45 min) then centrifuged (6000×g, 10 min, 4 °C) to collect soluble and insoluble material. The insoluble material was resuspended in 50 μL dH_2_O and all samples analysed via SDS-PAGE.

### ***A. caviae*** PseF^40^

*A. * caviae PseF (WP_139737850.1) pET-28a recombinant plasmid (purchased from GenScript, restriction enzymes NdeI and EcoRI) was electroporated into *E. coli* BL21(DE3) cells, streaked onto LB_Kan_ agar and incubated (37 °C, overnight). For expression trials, a single colony was used to inoculate LB_Kan_ (60 mL) and incubated (30 °C, 180 rpm, overnight) before being diluted in 2xYT_Amp_ to an OD_600_ of 0.02 and incubated (180 rpm, 37 °C). Expression trials were conducted by subjecting aliquots of culture to different induction conditions and cell pellets collected via centrifugation (10,000×*g*, 10 min, 6 °C). Cell pellets were resuspended in BugBuster containing protease inhibitor tablets and 1 mg mL^−1^ lysozyme and treated as per the manufacturer’s instructions. Insoluble material was separated following centrifugation (10,000×g, 10 min, 6 °C) then resuspended in 50 μL dH_2_O and all samples analysed via SDS PAGE.

Following expression trials, a single colony of *E. coli* BL21(DE3) cells transformed with *Ac*PseF was used to inoculate LB_Kan_ (60 mL) and incubated (30 °C, 180 rpm, overnight). The culture was further diluted with LB_Kan_ (3 L) and incubated (37 °C, 180 rpm) until an OD_600_ of 0.6 was reached whereby IPTG was added to a final concentration of 0.1 mM and further incubated (30 °C, 180 rpm, 3 h). Cell pellets were collected via centrifugation (6000×*g*, 30 min, 4 °C), resuspended in cold lysis buffer supplemented with 1 mM MgCl_2_ and 10 mM β-mercaptoethanol and sonicated. The supernatant following centrifugation (17,700×*g*, 40 min, 4 °C) was loaded onto a HisTrap HP Ni^2+^ affinity column pre-equilibrated with 50 mM sodium phosphate buffer, pH 7.3, 400 mM NaCl, 1 mM MgCl_2_, 10 mM β-mercaptoethanol, and 10 mM imidazole. After washing (10 C.V) with the same buffer a linear gradient of 10 mM to 500 mM imidazole was applied (40 C.V) and fractions were analysed via SDS PAGE (Supplementary Fig. SI.3).

### ***A. caviae*** PseF characterisation^40^

Aliquots of *Ac*PseF were further purified by gel filtration in 25 mM Tris–HCl pH 7.3 buffer containing 50 mM NaCl and 2 mM MgCl_2_. Following SDS PAGE analysis, pure protein was extracted from bands at the expected PseF construct molecular weight and subject to trypsin digest. The resultant peptides were analysed by MALDI-MS and MS/MS and spectral data was compared to the Mascot database to identify the protein as *Ac*PseF (Supplementary Fig. SI.4).

Following gel filtration, aliquots of *Ac*PseF were dialysed into 25 mM sodium phosphate buffer pH 7.4 and analysed by circular dichroism at 30 °C, from 180 to 260 nm at a final concentration of 0.2 mg mL^−1^. Under these conditions 86.5% of *Ac*PseF was predicted to have a fixed secondary structure, suggesting that it is amenable for crystallisation studies (Supplementary Fig. SI.5). Secondary structure predictions were made from circular dichroism data using K2D3 (http://cbdm-01.zdv.uni-mainz.de/~andrade/k2d3/).

Aliquots of *Ac*PseF were dialysed into 20 mM Tris pH 7.8 buffer containing 50 mM NaCl and 2 mM MgCl_2_ and concentrated to 4 mg mL^−1^. 100 µL samples were applied to a Superdex S200 size-exclusion column (G.E. Healthcare) pre-equilibrated with the same buffer, attached to a system comprising of a Wyatt HELEOS-II multi-angle light scattering detector and a Wyatt rEX refractive index detector linked to a Shimadzu HPLC system (SPD-20A UV detector, LC20-AD isocratic pump system, DGU-20A3 degasser and SIL-20A autosampler). A 2.5 mg mL^−1^ BSA sample was run as a standard and all data analysed using Astra V software (Supplementary Fig. SI.6).

Reaction mixtures containing 130 μg mL^−1^ *Ac*PseF, 0.5 mM Pse5Ac7Ac (Sussex Research), 1.5 mM CTP, 1 mM MgCl_2_, 50 mM NaCl, 25 mM sodium phosphate, pH 7.4, were incubated at 25 °C, alongside control reactions with reaction mixture as described without either Pse5Ac7Ac **1**, CTP or *Ac*PseF. Reactions were analysed by-ESI LC–MS and a peak indicating the formation of CMP-Pse5Ac7Ac **3** ([M−H]^−^ 638.2) was only observed in the reaction containing all components thus suggesting *Ac*PseF functions as a CMP-Pse5Ac7Ac synthetase. However a peak corresponding to Pse5Ac7Ac **1** was also observed, even after 6.5 h indicating that the reaction had not gone to completion and/or that hydrolysis of CMP-Pse5Ac7Ac **3** was occurring (Supplementary Fig. SI.7).

### PseB and PseC activity assays

PseB 25 µM and PseC 25 µM were added to reaction mixtures to give final concentrations of 1 mM UDP-GlcNAc, 10 mM l-Glu and 1.5 mM PLP in 50 mM Tris–HCl pH 7.4, and incubated (120 rpm, 37 °C). Reaction progression was monitored by -ESI LC–MS over 6 h. PseB and PseC were dialysed into deuterated Tris–HCl buffer (50 mM, pH 7.4) and added to reaction mixtures to give final concentrations of 1 mM UDP-GlcNAc, 10 mM l-Glu and 1.5 mM PLP in deuterated 50 mM Tris-HCl buffer pH 7.4. PseB was added to a final concentration of 25 µM and PseC to a final concentration of 25 µM or 125 µM and the reactions incubated (120 rpm, 37 °C). Reaction progression was monitored by -ESI LC–MS over 2 h.

### PseH activity assays

PseB 25 µM, PseC 125 µM and PseH 50 µM were added to reaction mixtures to give final concentration of 1 mM UDP-GlcNAc, 10 mM l-Glu and 1.5 mM PLP in 50 mM sodium phosphate buffer pH 7.4, with varying concentrations of Ac-CoA (0 mM or 0.15 mM) or CoA (0 mM, 0.15 mM, 0.015 mM or 0.0015 mM) and acetylthiocholine iodide (0 mM, 2 mM, 20 mM or 100 mM). Reactions were incubated (120 rpm, 37 °C) and monitored by ESI LC–MS over 3 h (Supplementary Fig. SI.8).

### Chemoenzymatic synthesis and characterisation of CMP-Pse5Ac7Ac 3

A reaction mixture containing 2 mM UDP-GlcNAc **4** (90 mg), 0.0015 mM coenzyme-A **18**, 100 mM *S*-acetyl thiocholine iodide **16**, 4 mM pyridoxal 5′-phosphate, 20 mM l-glutamic acid, 3 mM phosphoenolpyruvate, 0.2 mg mL^−1^ PseB, 0.4 mg mL^−1^ PseC, 0.2 mg mL^−1^ PseH, 0.2 mg mL^−1^ PseG and 0.2 mg mL^−1^ PseI in 50 mM sodium phosphate pH 7.4 (total volume 74 mL), was incubated (37 °C, 12 h) and was monitored via ESI LC–MS for the production of Pse5Ac7Ac **1**. After 12 h, 0.2 mg mL^−1^ PseF, 4 mM CTP and 20 mM MgCl_2_ were added and the reaction was incubated (37 °C, 4 h) and monitored by ESI LC–MS for production of CMP-Pse5Ac7Ac **3**. The mixture was lyophilised, resuspended in 1:1 dH_2_O:EtOH and stored at 4 °C for 30 min to precipitate enzymes which were then removed via centrifugation (38,759×*g*, 1 h, 4 °C). The supernatant was diluted in dH_2_O before lyophilisation then resuspended in dH_2_O and passed through a 45 µM Millex syringe filter (Merck) before being applied to a 500 mL column packed with Bio-Gel P-2 resin (Bio-Rad) in HPLC-grade H_2_O at a flow rate of 30 mL/h. 4 mL fractions were collected for 24 h and analysed via ESI-LC–MS for the presence **3** before being pooled and lyophilised to afford **3** as a colourless foam (39 mg).

^1^H NMR (500 MHz, D_2_O) δ 8.02 (dd, *J* = 7.5, 1.0 Hz, 1H, H2′), 6.14 (d, *J* = 7.5 Hz, 1H, H1′), 6.00 (d, *J* = 4.1 Hz, 1H, H3′), 4.37 – 4.32 (m, 2H, H4′, H6′), 4.32–4.28 (m, 2H, H5, H6), 4.27–4.22 (m, 4H, H4, H5′, H7a′, H7b′), 4.15–4.09 (m, 1H, H8), 4.05–4.01 (m, 1H, H7), 2.25–2.20 (m, 1H, H3eq), 1.99 (s, 3H, 7NHAc), 1.96 (s, 3H, 5NHAc), 1.61 (ddd, *J* = 13.4, 12.0, 5.2 Hz, 1H, H3ax), 1.20 (d, *J* = 6.5 Hz, 3H, 9CH_3_). ^13^C NMR (126 MHz, D_2_O) δ 174.5, 173.2, 170.4, 165.8, 157.3, 141.7, 100.0, 96.5, 89.2, 82.9, 74.1, 72.7, 69.2, 68.6, 64.9, 64.38, 53.7, 48.8, 36.0, 22.0, 21.9, 17.2. HR-MS data was collected with –ESI MS: Expected [M−H]^−^ *m/z*: 638.1789, measured [M−H]^−^ *m/z*: 638.1727. ATR-FTIR V_max_: 3227, 2941, 2888, 1592, 1403, 1075 cm^−1^.

## Supplementary Information

Supplementary Information.

## Data Availability

All data generated and/or analysed in this study are included in this published article (and its Supplementary Information).
