# Supplementary material for: Reconstitution and optimisation of the biosynthesis of bacterial sugar pseudaminic acid (Pse5Ac7Ac) enables preparative enzymatic synthesis of CMP-Pse5Ac7Ac
Source: Sci Rep. 2021 Feb 26;11:4756. doi: 10.1038/s41598-021-83707-x (PMC7910423; doi:10.1038/s41598-021-83707-x)
Supplement: Supplementary file 1 — Supplementary Information. [file 41598_2021_83707_MOESM1_ESM.docx]

Supporting Information for:

*Reconstitution and optimisation of the biosynthesis of bacterial sugar pseudaminic acid (Pse5Ac7Ac) enables preparative enzymatic synthesis of CMP-Pse5Ac7Ac*

Harriet S. Chidwick,^a^ Emily K. P. Flack,^a^ Tessa Keenan, ^a^ Julia Walton, ^a^ Gavin H. Thomas,^b^ and Martin A. Fascione*^a^

^a^ Department of Chemistry, University of York, Heslington Road, York, YO10 5DD

^b^ Department of Biology, University of York, Heslington Road, York, YO10 5DD

*Corresponding author. Email: [martin.fascione@york.ac.uk](about:blank)

Contents

***C. jejuni* PseB, C, H, G, I expression trials............................................................................................3**

**PseF purfication ………………………………………………………………………………………………………………………………4**

**AcPseF characterisation ………………………………………………………………………………………………………………….5**

**PseB mechanism……………………………………………………………………………………………………………………………..7**

**PseC mechanism……………………………………………………………………………………………………………………………..8**

**PseH assays..…………………………………………………………………………………………………………………………………..9**

**CMP-Pse5Ac7Ac 3 NMR……………………………………………………………………..……………………………………….….10**


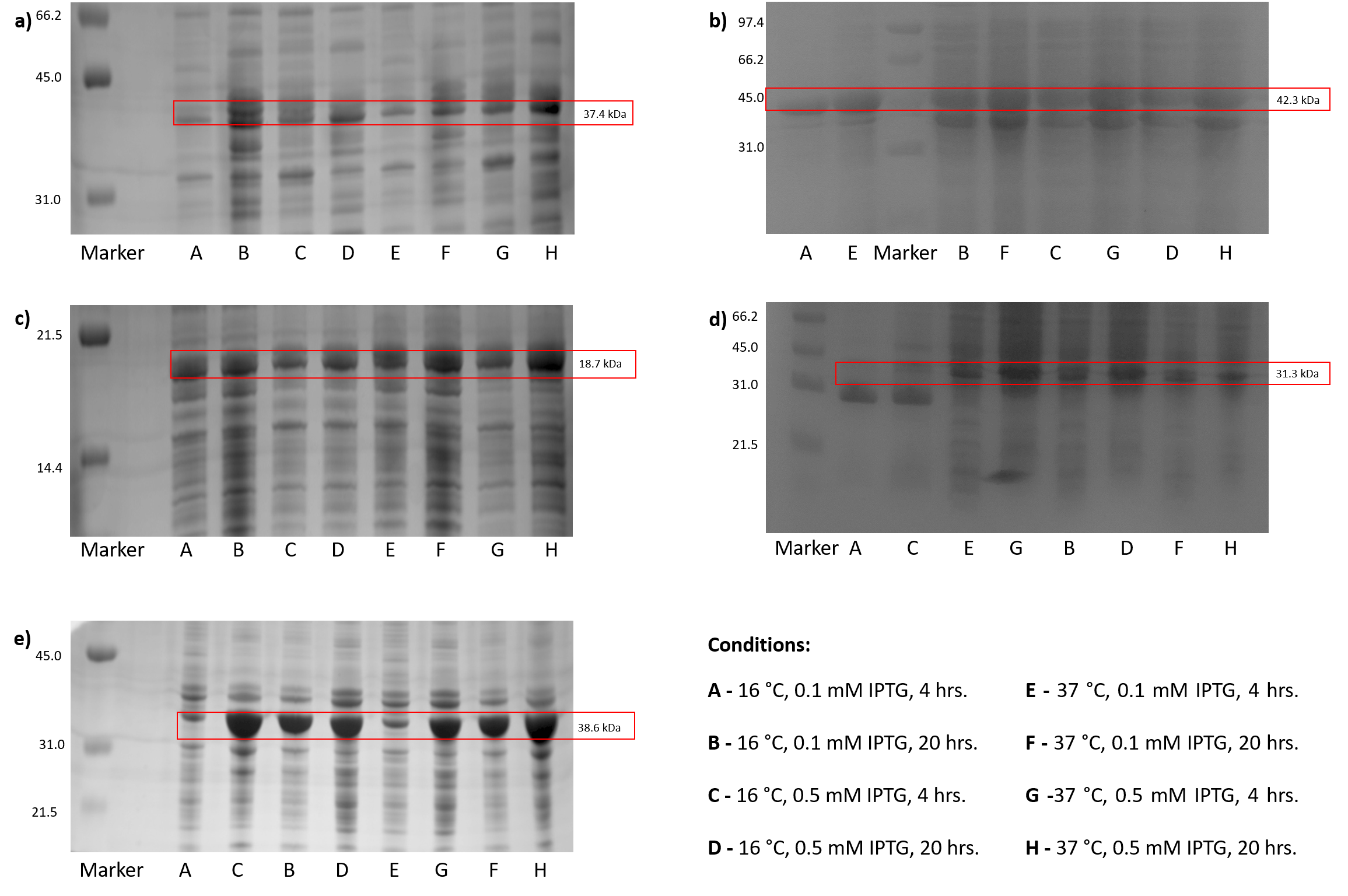


**Figure SI.1** Expression trials of the *C. jejuni* Pse5Ac7Ac biosynthetic enzymes screening different induction conditions;

**a)** PseB**, b)** PseC, **c)** PseH, **d)** PseG, and **e)** PseI.


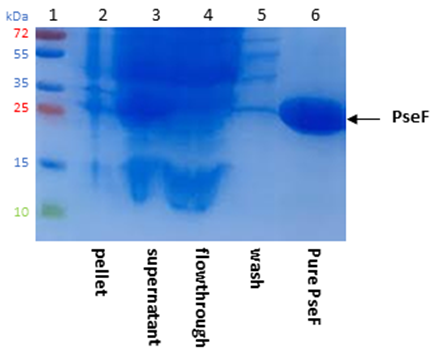


**Figure SI.3** SDS-PAGE analysis of *Ac*PseF following Ni^2+^-His_6_ purification; 1- PAGE Ruler MW Marker, 2- pellet, 3- supernatant, 4- flowthrough, 5- wash, 6- elution fraction.

PseF (MW 28.0 kDa) is present in the insoluble pellet, supernatant and as purified protein.


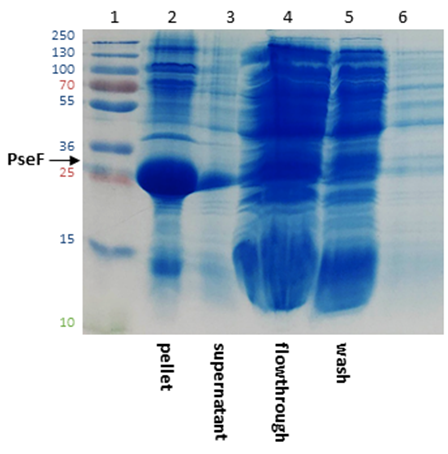


**Figure SI.2** SDS-PAGE analysis of *Hp*PseF following Ni^2+^-His_6_ purification; 1- PAGE Ruler MW Marker, 2- pellet, 3- supernatant, 4- flowthrough, 5- wash, 6- representative fraction.

*Hp*PseF (MW 28.0 kDa) is present almost exclusively in the insoluble pellet sample.


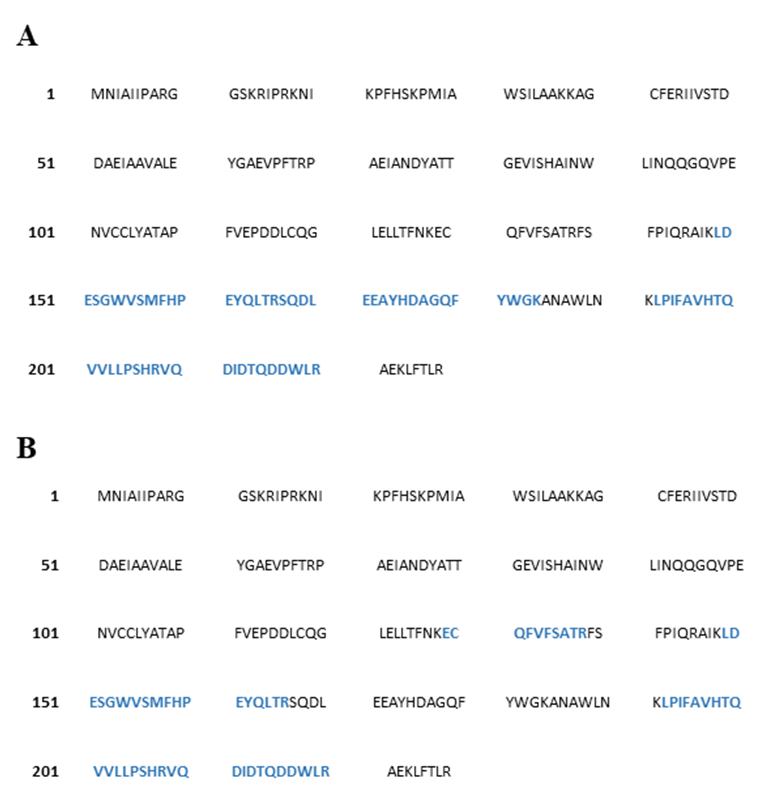


**Figure SI.4** Protein Identification Mass Spectrometry of *Ac*PseF following a tryptin digest, peptides identified are shown in blue text.


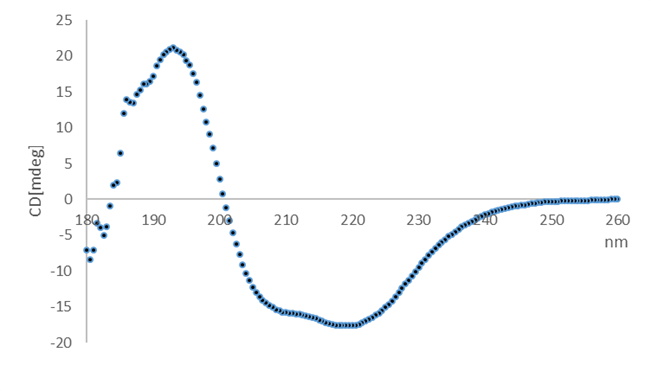


**Figure SI.5** *Ac*PseF 0.2 mg mL^-1^ circular dichroism spectrum at 30 °C in 25 mM sodium phosphate buffer pH 7.4.

**Figure SI.6** SEC-MALS characterisation (solid red refractive index, dashed red light scattering, and dotted red ultraviolet) of *Ac*PseF with a BSA standard. The peak $\sim$30 mins is consistent with a molecular weight of 54 kDa which corresponds to PseF existing predominantly as the homodimer, the minor peak at peak $\sim$27 mins is at very low intensity (<0.6% of the total) but is consistent with a molecular weight of 100 kDa which could either be a contaminant or correspond to further PseF aggregation existing as a dimer of dimers.


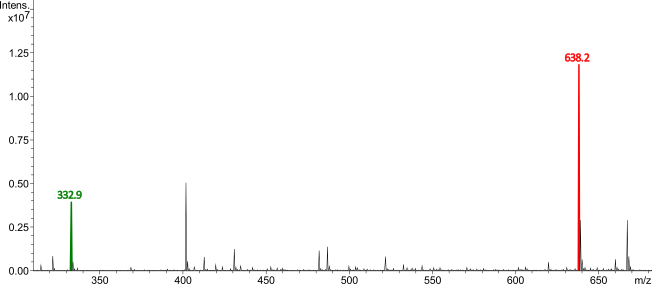


**Figure SI.7** -ESI LC-MS analysis showing *Ac*PseF catalysed conversion of Pse5Ac7Ac **1** ([M-H]^-^ 332.9) to CMP-Pse5Ac7Ac **3** ([M-H]^-^ 638.2) after 6.5 hours.

*PseB mechanism in deuterated buffer*


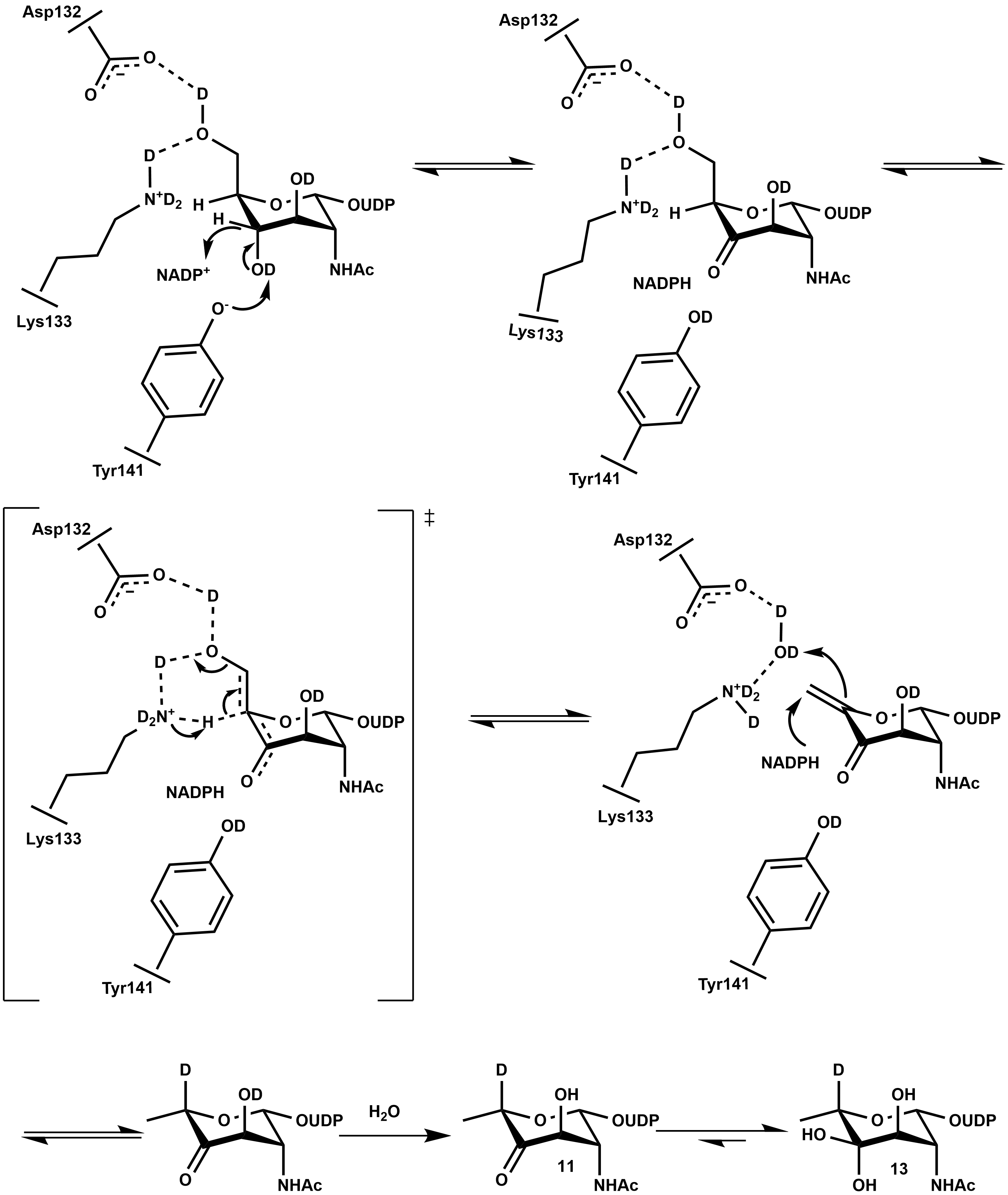


**Scheme SI.1** Proposed PseB mechanism in deuterated buffer depicting the formation of a C-D bond in the product and the expected exchange of labile deuterium and protons during ESI LC-MS analysis to afford 11.

*PseC mechanism in deuterated buffer*


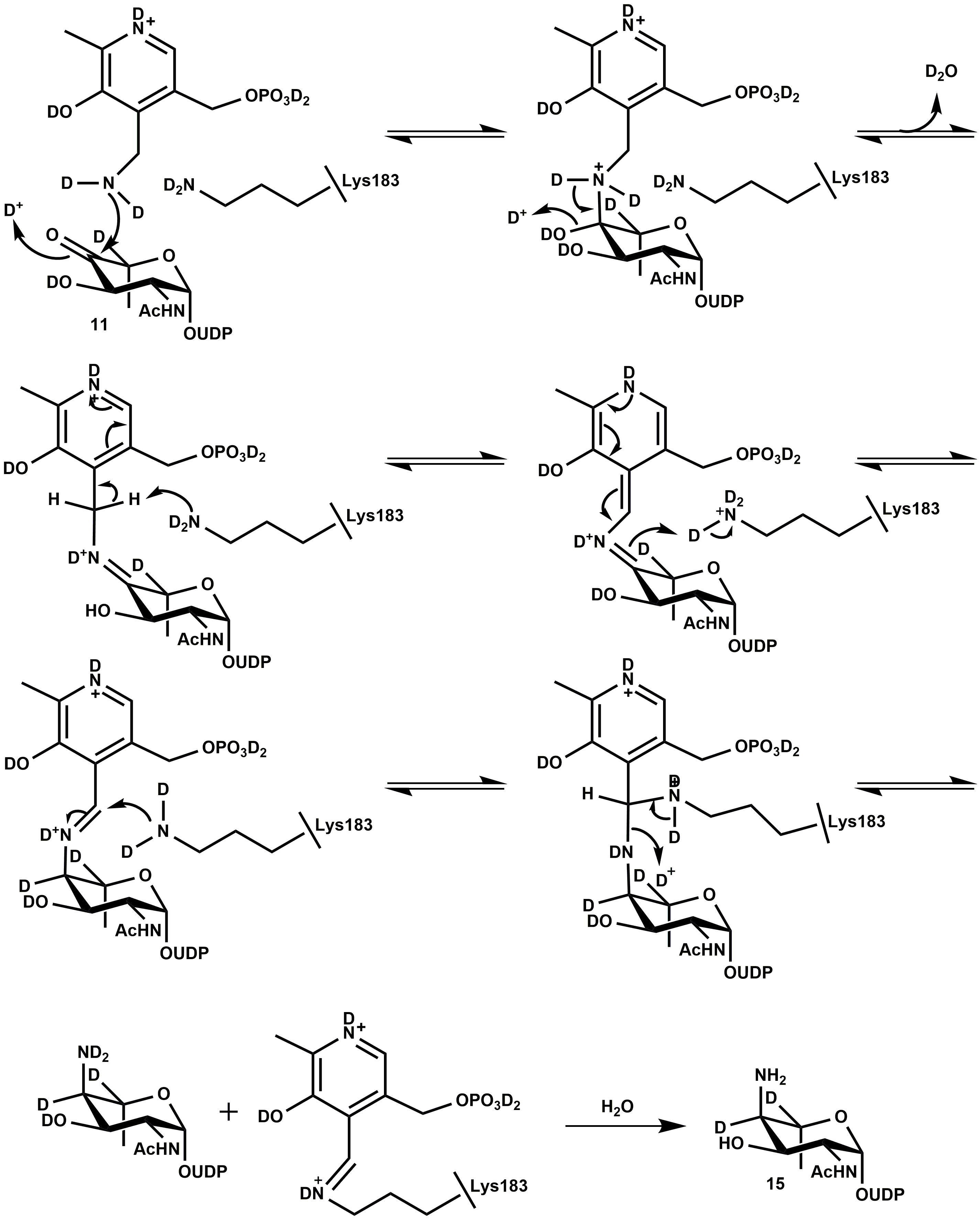


**Scheme SI.2** Proposed PseC mechanism in deuterated buffer depicting the formation of a C-D bond in the product and the expected exchange of labile deuterium and protons during ESI LC-MS analysis to afford **15**.

**Figure SI.8** LC-MS negative ESI mass spectra demonstrating conversion to the acetylated PseH product **17** 0.0015 mM CoA **18** with acetylthiocholine iodide **16** as a regeneration factor at either **a)** 20 mM, or **b)** 100 mM.


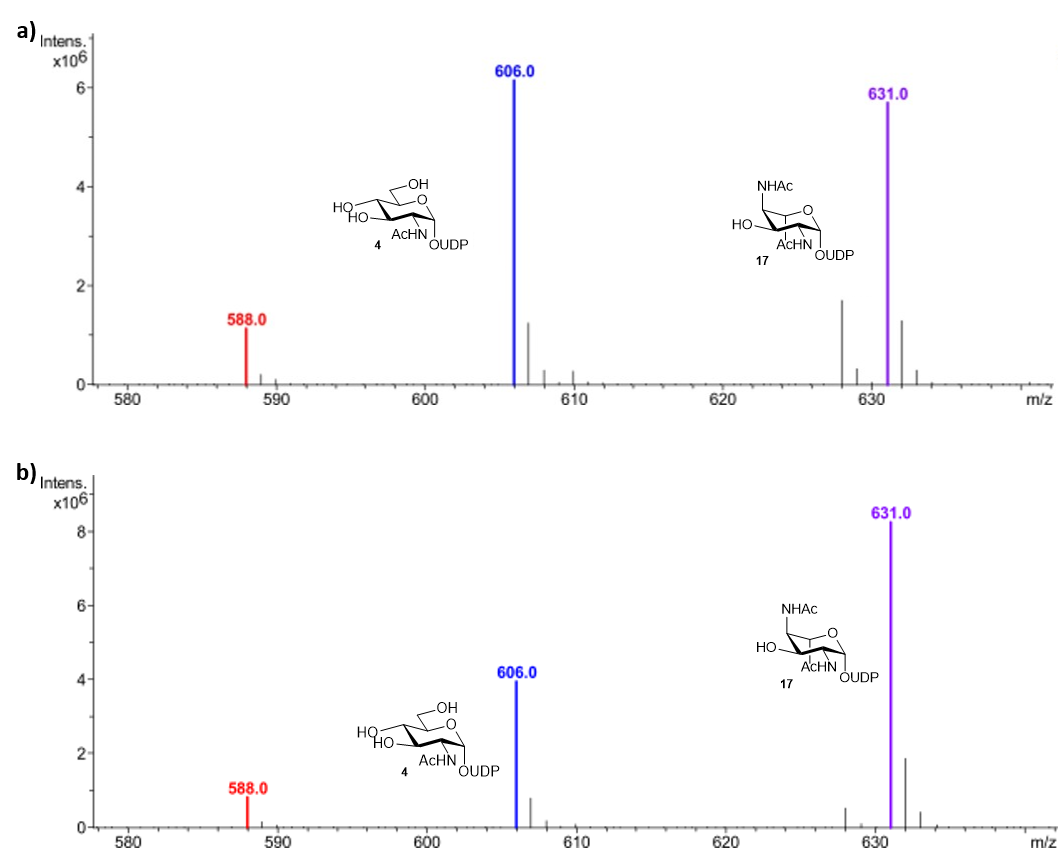


^^

**Figure SI.9** ^1^H NMR of CMP-Pse5Ac7Ac **3**.

**Figure SI.10** HSQC NMR of CMP-Pse5Ac7Ac **3**.
